# Andiroba oil: a strategic approach to detect antioxidant activity in different lipid groups by TLC

**DOI:** 10.1186/1753-6561-8-S4-P233

**Published:** 2014-10-01

**Authors:** Hugo Salgado, Alberdan Santos

**Affiliations:** 1 Federal Universityof Pará, Belém, PA, Brazil

## 

The search for natural sources of molecules that exhibit biological activity has intensified in the last 20 years. The andiroba (Carapa guianensis) oil is known for its medicinal actions [1], and biological activities, among them the antioxidant action [2]. The use of traditional techniques for the determination of antioxidant activity in vegetable oils faces strong competition from modern techniques and more updated, but the thin layer chromatography (TLC) is presented as effective, quick, simple and low cost. In this study we applied the technique chromatographic separation of lipid groups in the crud andiroba oil [3] in order to identify groups that have antioxidant activity [4]. The crud andiroba oil used was purchased at a market in Belém / Pa. The oil was applied on TLC plate (10x20cm) eluted with a solvent system consisting of hexane: diethyl ether: acetic acid (80:20:2) to afford five fractions. The developer DPPH was applied on the plate containing lipid groups separated to reveal which fractions had antioxidant activity. The results showed that the five fractions obtained after the separation in TLC were identified by their Rf's as phospholipid (0.012) monoacylglycerols (0.062), diacylglycerols (0.25), free fatty acids (0.437), triglycerides (0.75) and cholesterol esters (0,87). After applying of developer, the groups that showed characteristic coloration of activity antioxidant by DPPH were monoacylglycerols, diacylglycerols, triacylglycerols and free fatty acids. The other fractions showed no antioxidant activity. These results showed antioxidant activity in the fractions separated by TLC, showing which groups of lipids present are responsible for this activity in crude andiroba oil.
